# Association of NFKB1 gene polymorphism (rs28362491) with cardiometabolic risk factor in patients undergoing coronary angiography

**DOI:** 10.34172/jcvtr.2023.31834

**Published:** 2023-09-23

**Authors:** Zahra Darabi, Sara Jambarsang, Mohammad Yahya Vahidi Mehrjardi, Seyed Mostafa Seyed Hosseini, Mohammadtaghi Sarebanhassanabadi, Mahdieh Hosseinzadeh, Sara Beigrezaei, Azam Ahmadi Vasmehjani, Marzieh Taftian, Vahid Arabi, Maryam Motallaei, Faezeh Golvardi Yazdi, Amin Salehi-Abargouei, Azadeh Nadjarzadeh

**Affiliations:** ^1^Department of Nutrition, School of Public Health, Shahid Sadoughi University of Medical Sciences, Yazd, Iran; ^2^Research Center for Food Hygiene and Safety, School of Public Health, Shahid Sadoughi University of Medical Sciences, Yazd, Iran; ^3^Center for Healthcare Data Modeling, Departments of Biostatistics and Epidemiology, School of Public Health, Shahid Sadoughi University of Medical Sciences, Yazd, Iran; ^4^Department of Genetic, Shahid Sadoughi University of Medical Sciences, Yazd, Iran; ^5^Yazd Cardiovascular Research Center, Non-Communicable Diseases Research Institute, Shahid Sadoughi University of Medical Sciences, Yazd, Iran; ^6^Department of Nursing, School of Nursing and Midwifery, Shahid Sadoughi University of Medical Sciences and Health Services, Yazd, Iran

**Keywords:** NFKB1 polymorphism, Cardiovascular disease, Lipid profile, Liver enzymes

## Abstract

**Introduction:**

Genetic and environmental factors are involved in the pathogenesis of cardiovascular diseases (CVDs). The aim of the study was to investigate between the genotype of the NFKB1 gene and the cardiometabolic risk factor in patients undergoing coronary angiography.

**Methods:**

This cross-sectional study was conducted on 462 adults (male and women) aged between 35 and 75 years who referred to Afshar Hospital for coronary angiography in 2021- 2022. The polymerase chain reaction restriction fragment length polymorphism method was used to detect the genotype of rs28362491. Biochemical parameters were measured using commercial kits. Gensini and Syntax scores were calculated using the angiography result to assess the extent of coronary artery stenosis. We used multivariate logistic regression analysis to examine the relationship between genotype variants and cardiometabolic risk factors.

**Results:**

There was no association between variant genotypes and abnormally levels of serum alanine aminotransferase (ALT) (*P* value=0.51), aspartate aminotransferase (AST) (*P* value=0.99), triglyceride (TG) (*P* value=0.48), total cholesterol (*P* value=0.79), low density lipoprotein-cholestero (LDL-C) (*P* value=0.31), high-density lipoprotein-cholesterol (HDL-C) (*P* value=0.53), fast blood sugar (FBS) (*P* value=0.39), systolic blood pressure (*P* value=0.14), diastolic blood pressure (*P* value=0.64), Gensini score (*P* value=0.48) and syntax score (*P* value=0.74) in the crude model even after adjustment for confounding factors.

**Conclusion:**

We found no association between the ATTG polymorphism and cardiometabolic risk factors in patients who had coronary angiography. Further investigations are needed to assess the association between variants of 28362491 and cardiometabolic markers.

## Introduction

 According to the World Health Organization (WHO), CVDs are the leading cause of death globallyy, accounting for an estimated 17.8 million deaths in 2017, representing 33% of all global deaths.^1^ CVDs are multifactorial diseases. The risk of cardiovascular diseases is increased by lower levels of physical inactivity, type 2 diabetes, a Western diet, high blood pressure, obesity, and dyslipidemia.^2,3^ CVDs are affected by age, genetic heritage, and gender as invariable.^4^

 CVD mainly results from atherosclerosis, which is the build up of plaque in the arteries.^5^ Inflammation, both systemic and local, is a key factor in the onset and advancement of atherosclerosis, affecting the endothelium and leading to clinical manifestations.^6,7^ Inflammatory biomarkers can indicate CVD risk, regardless of other risk factors.^7^ Chronic inflammatory markers such as C-reactive protein (CRP), interleukin 1 (IL-1), tissue necrosis factor-a (TNF-a), and interleukin-6 (IL-6) are higher in patients with dyslipidemia and diabetes.^8,9^ Increased aminotransferase levels can also cause higher levels of inflammation and oxidative stress markers, which predict chronic disease-related mortality and future cardiovascular diseases.^10,11^ This means that oxidative stress and inflammation, which can lead to CVDs, are enhanced by high levels of TG, LDL-C, total cholesterol, FBS, ALT and AST and low level of HDL-C.^12^

 Genetics is also an important factor in the development and progression of CVDs. Previous studies have demonstrated that single nucleotide polymorphisms (SNPs) are associated with CVDs and their risk factors.^13,14^ The protein p50 is coded by the NFB1 gene on chromosome 4q24. The -94 ins/del ATTG (rs28362491) polymorphism is an insertion/deletion of a 4 base pair ATTG sequence at position -94.^15,16^ The genetic mutation affected the synthesis of p50. The deletion of an ATTG in the promoter region of the NFKB1 gene reduced the synthesis of p50.^16^ Some studies suggest that people with the DD genotype or the rs28362491-D allele have a higher risk of inflammatory diseases such as CVD.^13,17,18^ Si-Yu Jin et al found that the DD genotype of the NFKB1 gene increased the risk and severity of acute coronary syndrome in the Han population in China.^13^ The relation between the rs28362491 genotype and CVDs risk factors such as blood glucose, lipid profile, and liver enzymes has been studied less. Yenmis et al reported that the NF-B1 promoter regulatory gene is associated with morbid obesity and high levels of alkaline phosphatase (ALP), alanine ALT, AST, and gamma-glutamyltransferase (GGT).^14^ Genotype II of rs28362491 predicted the risk and severity of type 2 diabetes-associated dyslipidemia.^9^

 A meta-analysis study published in 2020. Its results show that race can affect the association between this polymorphism and the risk of coronary artery disease.^19^ Rs28362491 variants were significantly associated with increased coronary artery disease risk in Asians under five genetic models. For the Caucasian population, only weak positive associations were seen in the heterozygous and dominant models, and no association was found under other contrasts.To our best knowledge, no study has investigated the association between this polymorphism and the risk factors of cardiovascular disease in IRAN. We aimed to assess blood pressure, fasting blood sugar, lipid profile, liver enzymes, atherosclerosis indices, and rs 28362491 variants in the Iranian population.

## Materials and Methods

###  Study population

 We performed a cross-sectional study on 462 patients who underwent coronary angiography at Afshar Hospital in Yazd city, IRAN in 2021-2022. We included adults aged 35 to 75 years who had coronary angiography and consented to participate in the study.

 The exclusion criteria were autoimmune diseases, cancer, kidney or liver failure, coronary artery disease bypass grafting (CABG), history of percutaneous coronary intervention (PCI), pregnant or lactating women, and individuals with a BMI > 40 kg/m2. All participants were consented written informed for entering and publication of study results. This study was approved by the Ethics Committee of Shahid Sadoughi University of Medical Sciences, Yazd, Iran (IR.SSU.SPH.REC.1400.108).

###  Calculation of sample size

 The sample size was calculated using the QUANTO program version 1.2.4 [α = 5% and power (1-β) = 80%]. The estimated sample size was 429 people. We included 462 people to account for possible dropouts.

###  Extraction of DNA and Single-nucleotide polymorphism genotyping 

 To extract genomic DNA from peripheral vein blood leukocytes, a whole blood genome extraction kit (SIMBIOLAB, IRAN) was used according to the instructions of the manufacturer. Until analysis, the DNA samples were kept at -20°C. To detect the genotype, the polymerase chain reaction-restriction fragment length polymorphism (PCR-RFLP) method was utilized. The primers for amplifying the genomic DNA fragment are 5′-TGGGCACAA GTCGTTTATGA-3′ and 5′-CTGGAGCCGGTAGGGAAG-3′. The PCR protocol was a primary temperature of 94℃ (5 min) after 35 amplification cycles (30 s at 94℃, 45 s at 59℃, and extension at 72℃ for 1 min). The ultimate extension step was performed at 72℃ for 2-min. One unit of restriction enzyme PfIMI (Van91I, 10 U/μL, Fermentas International Inc, Canada) was used to digest PCR product for detecting two different alleles, the 285 bp (D allele) and 45 bp and 240 (I allele). By incubation of the PCR product–enzyme mix overnight at 37 °C, it was run finally on electrophoresis at 90 V for 45 min in 2% agarose gel. Three DNA fragments were represented with various lengths of heterozygous DI (3 bands: 285 & 240 & 45 bp), homozygous II (2 bands: 45 &240 bp), and homozygous DD (1 band: 285 bp).

####  Biochemical assessment

 Blood samples (4 mL) were taken from all participants after fasting overnight. The samples were collected in EDTA-containing tubes for biochemical assays. The samples were centrifuged for 5 min at 5000 rpm for the separation of plasma from blood cells. For further analysis, serums were stored at 80 °C separately. Commercial kits were used to measure total cholesterol(Cat No BXC0312C), Glucose (Cat No BXC0312C), TG, HDL-C (Cat No BXC0315B) and LDL-C(Cat No BXC0315B) (Biorex fars, Iran), and ALT, AST (Pars Azmun, Karaj, Iran). The abnormal higher serum liver enzyme levels were determined in terms of NHANES definition as levels of > 30 IU/L for females and ALT > 47 IU/L for males; AST > 33 IU/L in women and men.^20^ Total cholesterol above 200 mg/dL was determined as hypercholesterolemia. Moreover, LDL-C above 100 mg/dL was abnormal.^21^ In the present work, patients were classified as high TG and normal TG. The TG level higher than 150 mg/dl was hypertriglyceridemic. The subjects with fasting glucose levels > 126 mg/dL were also abnormal.^22^

####  Calculation of Syntax and Gensini score

 To calculate Syntax and Gensini scores, the angiography result was considered to assess the coronary stenosis extent. The Syntax score was estimated using a computer program such as interactive and sequential self-guided questions. The syntax score algorithm is accessible on the Syntax website (www.syntaxscore.com).^23^ To calculate the Gensini score, a severity score was given to each coronary stenosis as 1 point for ≤ 25% narrowing, 2 points for 26 to 50% narrowing, 4 points for 51 to 75% narrowing, 8 points for 76 to 90% narrowing, 16 points for 91 to 99% narrowing, and 32 points for total occlusion. Then, each lesion score was multiplied by a factor considering the importance of the lesion’s position in the coronary circulation. For each patient, the total Gensini score was the summation of each person based on the associated stenotic artery.^24^

###  Assessment of other variables

 General demographic data included age, medical history, smoking status, and use of medicine, which were gathered by experienced interviewers. To assess the physical activity of the patients, International Physical Activity Questionnaire (IPAQ) was used.^25^ Physical activity level was determined based on metabolic equivalent task (MET) min per week. A nurse determined the blood pressure of the patient.

 Higher blood pressure was determined as Systolic blood pressure ≥ 140 mm Hg or diastolic blood pressure ≥ 90 mm Hg.^26^

###  Statistical analysis

 To perform statistical analysis, SPSS 23.0 was used. The Chi-squared and one-way ANOVA were utilized for assessing the categorical and continuous variables in genotype variants. The independent contribution of genotypes to risk factors of CAD was assessed through multivariate logistic regression analysis in crude and multivariable-adjusted models. Adjustment for confounding variables including BMI, physical activity, age and gender was performed in the first model. Additional adjustment for smoking and medication use was done in the second model. For each odds ratio (OR), confidence intervals of 95% were calculated. In all cases, *P* < 0.05 was a significant difference statistically.

## Results

###  General characteristics study participants

 The mean age of the participants was 57.03 years. Frequencies of ins/ins, ins/del and del/del genotypes were %31.7, %52.9 and %18.5 respectively in population. General characteristics of the participants across genotype rs28362491 are demonstrated in Table 1. Physical activity, medication use, BMI, gender and smoking were not different among genotypes.

**Table 1:** General characteristics of the participants across genotype rs28362491

	**Genotype II**^1^ **(132)**	**Genotype DI**^2^ **(247)**	**Genotype DD**^3^ **(83)**	***P*** value**
BMI(kg/m^2^)*	26.86 ± 4.08	27.79 ± 4.21	27.06 ± 4.70	0.11
Age (year)*	57.40 ± 9.78	56.01 ± 9.24	59.30 ± 8.52	0.02
Gender, male, n (percent)	78 (59.5)	155(62.8)	57(68.7)	0.40
Smoking, n(percent)				0.34
Non smoker	82(62.6)	161(65.7)	53(63.9)	
Former smoker	3(2.3)	8(3.3)	6(7.2)	
Current smoker	46(35.1)	76(31)	24(28.9)	
Physical activity (min per week)*	4111.25 ± 7389.62	4264.86 ± 7518.39	3805.36 ± 6747.85	0.88
Medication use, yes, n (percent)				
Statins	41(31.1)	93 (37.7)	30(36.1)	0.43
Anti hypertension	59(44.7)	106(42.9)	38(45.8)	0.88
Anti diabetes	42(31.8)	85 (34.4)	23(27.7)	0.52

BMI: Body mass index ^1^ NFKB1 gene rs28362491 (-94ATTGinsertion/insertion) ^2^ NFKB1 gene rs28362491 (-94ATTGinsertion/ deletion) ^3^ NFKB1 gene rs28362491 (-94ATTG deletion / deletion) *Data presented as mean ± SD **Obtained from one way Anova for continuous variables and Chi-squared test for categorical variables

###  Distribution risk factors in genotypes of rs28362491

 Biochemical parameters and other risk factors for CVD across variants of genotype are shown in Table 2. Levels of ALT, AST, LDL, HDL, TG were not significantly different between genotype. Other risk factor such as blood pressure also indices of atherosclerosis (gensini and syntax score) were not different between genotypes. Patients with DI genotype had higher total cholesterol compared to patients with DD and II genotype (*P* value = 0.04).

**Table 2:** Biochemical parameters and other risk factors for Cardiovascular disease across variants of genotype rs28362491

	**Genotype II**	**Genotype DI**	**Genotype DD**	***P*** value*
High ALT, n(percent)	24(32.9)	55(28.5)	14(27.5)	0.74
High AST, n(percent)	45(60.8)	119(61.7)	31(60.8)	0.98
High Total cholesterol, n(percent)	34(29.6)	96(40.5)	20(27.8)	0.04
High LDL, n(percent)	54(47)	105(44.3)	28(39.4)	0.60
Low HDL, n(percent)	37(32.2)	78(32.6)	26(36.6)	0.61
High TG, n(percent)	42(36.5)	88(37)	30(41.7)	0.74
High FBS, n(percent)	51(40.2)	95(40.1)	27(34.2)	0.61
High systolic blood pressure, n(percent)	28(22)	44(19)	11(13.8)	0.33
High diastolic blood pressure, n(percent)	23(18.1)	37(16)	10(12.5)	0.79
Hjgh Gensini score, n(percent)	57(47.6)	117(50.4)	40(50.6)	0.65
High Syntax score, n(percent)	23(18.4)	43(18.5)	16(20.3)	0.93

ALT: Alanine aminotransferase; AST: Aspartate aminotransferase; LDL-C, low density lipoprotein-cholesterol; HDL-C, high-density lipoprotein-cholesterol; TG, triglyceride; FBS: fast blood sugar * Obtained from Chi-squared test

###  Association between genotypes of rs28362491 and risk factors of CVD

 Multi-variable adjusted odds ratios (ORs) for risk factor of CVD across genotypes are represented in Table 3. Variants of genotypes were not associated with odds of developing abnormally high serum ALT (OR: 0.77; 95% CI: 0.35–1.69, *P* = 0.51), AST (OR: 0.99; 95% CI: 0.48–2.07, *P* = 0.99), TG(OR: 1.24; 95% CI: 0.67–2.26, *P* = 0.48), LDL(OR: 0.73; 95% CI: 0.40–1.34, *P* = 0.31), HDL(OR: 1.21; 95% CI: 0.65–2.26, *P* = 0.53), total cholesterol (OR:0.91; 95% CI: 0.47-1.76 *P* = 0.79) and FBS (OR:0.77; 95% CI: 0.43–1.38, *P* = 0.39) in crude model also after adjustment for confounding factor including of BMI, levels of physical activity, use medication, age, gender and smoking.

**Table 3:** Multi-variable adjusted odds ratios (ORs) for risk factor of Cardiovascular disease across genotypes rs28362491

**High ALT**	**Genotype II**	**Genotype DI**	**Genotype DD**	***P***** value¹**	***P***** trend**
Crude	1.00	0.81 (0.45_1.45)	0.77 (0.35_1.69)	0.51	0.48
Model1	1.00	0.63 (0.34_1.18)	0.73 (0.32_1.64)	0.45	0.35
Model2	1.00	0.57 (0.30_1.08)	0.65 (0.28_1.48)	0.30	0.23
High AST					
Crude	1.00	1.03 (0.59_1.79)	0.99 (0.48_2.07)	0.99	0.98
Model1	1.00	0.78 (0.42_1.42)	0.88 (0.38_1.82)	0.65	0.60
Model2	1.00	0.85 (0.46_1.56)	0.89 (0.40_1.96)	0.77	0.74
High TG					
Crude	1.00	1.02 (0.64_1.61)	1.24 (0.67_2.26)	0.48	0.52
Model1	1.00	0.97 (0.59_1.61)	1.25 (0.66_2.38)	0.48	0.54
Model2	1.00	0.92 (0.53-1.59)	1.52 (0.74-3.10)	0.24	0.33
High Total cholesterol					
Crude	1.00	1.62 (1.00_2.61)	0.91 (0.47_1.76)	0.79	0.85
Model1	1.00	1.56 (0.93_2.62)	0.84 (0.41_1.71)	0.63	0.95
Model2	1.00	1.45 (0.85_2.47)	0.76 (0.36_1.57)	0.46	0.72
High LDL-C					
Crude	1.00	0.89 (0.57_1.40)	0.73 (0.40_1.34)	0.31	0.32
Model1	1.00	0.85 (0.53_1.38)	0.73 (0.39-_1.38)	0.33	0.33
Model2	1.00	0.81 (0.50_1.33)	0.66 (0.34_1.27)	0.21	0.20
Low HDL-C					
Crude	1.00	1.02 (0.63_1.64)	1.21 (0.65_2.26)	0.53	0.57
Model1	1.00	0.99 (0.59_1.65)	1.16 (0.59_2.27)	0.65	0.69
Model2	1.00	1.03 (0.60_1.75)	1.26 (0.63_2.51)	0.50	0.53
High FBS					
Crude	1.00	0.99 (0.64_1.54)	0.77 (0.43_1.38)	0.39	0.61
Model1	1.00	1.00 (0.62_1.60)	0.79 (0.42_1.46)	0.45	0.90
Model2	1.00	0.94 (0.58_1.53)	0.78 (0.41_1.46)	0.44	0.46
High Gensini score	Q1	Q2	Q3		
Crude	1.00	1.21 (0.78_1.87)	1.22 (0.69_2.15)	0.48	0.42
Model1	1.00	1.18 (0.71_1.95)	1.00 (0.52_2.23)	0.99	0.89
Model2	1.00	1.15 (0.69_1.92)	1.04 (0.54_1.98)	0.90	0.83
High Syntax sore					
Crude	1.00	1.00 (0.57_1.76)	1.12 (0.55_2.29)	0.74	0.76
Model1	1.00	0.99 (0.53_1.83)	1.04 (0.49_2.23)	0.90	0.91
Model2	1.00	1.02 (0.55_1.90)	1.06 (0.49_2.27)	0.87	0.88
High Systolic blood pressure					
Crude	1.00	0.82 (0.48_1.41)	0.56 (0.26_1.20)	0.14	0.14
Model1	1.00	0.80 (0.46_1.40)	0.58 (0.26_1.26)	0.17	0.17
Model2	1.00	0.82 (0.46_1.45)	0.59 (0.27_1.30)	0.19	0.19
High Diastolic blood pressure					
Crude	1.00	0.86(0.48_1.52)	0.64(0.29_1.44)	0.64	0.29
Model1	1.00	0.91(0.49_1.69)	0.53(0.22_1.30)	0.16	0.20
Model2	1.00	0.96(0.51_1.78)	0.54(0.22_1.34)	0.18	0.23

ALT: Alanine aminotransferase; AST: Aspartate aminotransferase; LDL-C, low density lipoprotein-cholesterol; HDL-C, high-density lipoprotein-cholesterol; TG, triglyceride; FBS: fast blood sugar Model1: Adjusted for BMI, Physical activity, gender, age Model2: Additionally, adjustment for smoking and medication use (Statins, Anti hypertension, Anti diabetes) *P* value¹: Genotype DD compered to genotype II

## Discussion

 This paper reports no evidence of an association between the rs28362491 genotype and CVD risk among patients who underwent coronary angiographyPrevious studies have extensively investigated the -94 ins/del ATTG polymorphism in relation to cardiovascular diseases, but they have yielded inconsistent results. Coto et al found no significant difference in the frequencies of allele and rs28362491 genotype between the healthy group and CAD patients.^27^ Other studies have reported a significant association between rs28362491 and CAD. SY et al reported that patients with genotype DD had significantly higher Gensini scores than patients with genotype II in acute coronary syndrome (ACS).^13^

 The results of a case-control study revealed an association between SNP rs28362491 and CAD risk in a recessive model after adjusting for cardiovascular risk factors.^28^

 A meta-analysis study found that the mutant D allele in rs28362491 increased the odds of CAD.^19^

 The NFKB1 gene increased the expression of pro-inflammatory cytokines.^29^ The NFKB1-94ins/del ATTG polymorphism caused elevated IL-6 levels, suggesting a mechanistic link between rs28362491 and CAD susceptibility.^30^ However, this study found no association between the NFKB1-94ins/del ATTG polymorphism and the Gensini and Syntax scores. Previous studies have reported inconsistent findings. The different variant allele frequency of rs28362491 polymorphisms in the Iranian population, reflecting the different genetic background of Iran from other ethnic groups, may explain why our study did not replicate the genetic association with CAD risk found in other studies. For example, Hong-Mei La et al reported an association between SNP rs28362491 and CAD risk in a recessive model after adjusting for cardiovascular risk factors.^16^ However, this association was not observed in additive and dominant. In their study, the frequency of II genotype was 37.2% in patients and 41.2% in healthy group, while in our study it was 31.7%.

 We found no association between the rs28362491 genotype and the odds of higher FBS. Few studies have examined this relationship. One study reported that the frequency of genotype II for rs28362491 was significantly higher in non-diabetics than in diabetic patients.^31^ Our results showed that rs28362491 (-94 deletion) was not associated with the likelihood of higher liver enzyme and the levels of HDL, LDL and TG. Few studies have evaluated the association of rs28362491 genotypes with CVD risk factors such as total cholesterol, higher TG, and lower HDL. A study reported a significant association of NF-κB1 -94ins/del ATTG polymorphism with the risk of diabetes and diabetes-related dyslipidemia in the dominant and heterozygous genetic models.^9^ Fakhir et al found no significant association between NFKB1-94 ins/del ATTG genotypes and levels of AST and ALT.^32^ Yongchao et al conducted a case-control study to assess the association of genotype rs28362491 with hepatocellular carcinoma development in HBV-infected patients. They divided the patients into high (above 45 U/l) and normal ALT (below 45 U/l) groups and evaluated the association between the polymorphisms with hepatocellula r carcinoma risk. Genotype II was significantly associated with an increased risk of hepatocellular carcinoma in the normal ALT group.^33^

 Few studies have examined the association of this polymorphism with the risk factors of CAD and the evidence is inconclusive.

 We found no association between NFKB1-94Ins/Del ATTG genotypes and blood pressure. To our knowledge, no study has examined the association of blood pressure with this polymorphism, but a study found that the homozygous genotype II subjects had higher NOS3 protein expression than the homozygous genotype DD subjects under laminar shear stress.^34^ The expression of NOS3 may influence the blood pressure.^35^

 The mechanism of NFKB1-94 Ins/Del ATTG polymorphism on CVD risk factors is unclear. This gene encodes the p50 protein. An experimental study showed that fatty acid oxidation at the molecular, whole body, and tissue levels was higher in p50 knockout mice (p50 KO) than in wild-type mice. Furthermore, p50 KO mice were resistant to high-fat diet-induced obesity. These results suggest that NF-κB signaling regulates fatty acid utilization and affects obesity susceptibility.^36^

 This study had several limitations. Polymorphisms are a common form of genetic variation and one polymorphism has a small effect. More studies on SNPs are needed to assess the association of the NFKB1 gene with CVD risk factors in other races. Second, our results were limited to the Iranian population, and they may not generalize to other races. Third, this was a single-center study with a relatively small number of patients.

## Conclusion

 In the present study, no association was found between ATTG polymorphism and cardiometabolic risk factors in the Iranian population. Studies in this field should be done in different regions.

## Acknowledgements

 The authors would like to thank all participants without whom this study was impossible.

## Competing Interests

 There is no conflict of interest.

## Ethical Approval

 This study was approved by the Ethics Committee of Shahid Sadoughi University of Medical Sciences, Yazd, Iran ( IR.SSU.SPH.REC.1400.108).

## Funding

 The present study was supported by a grant provided by Shahid Sadoughi University of Medical Sciences.
